# Liesegang-like patterns of Toll crystals grown in gel

**DOI:** 10.1107/S0021889812051606

**Published:** 2013-02-14

**Authors:** Monique Gangloff, Abel Moreno, Nicholas J. Gay

**Affiliations:** a Department of Biochemistry, University of Cambridge, 80 Tennis Court Road, Cambridge CB2 1GA, UK; b Instituto de Química, Universidad Nacional Autónoma de México, Mexico DF 04510, Mexico

**Keywords:** Toll receptor, Liesegang rings, counter-diffusion capillary crystallization, malonate, magic triangle, I3C, Ostwald ripening, repetitive patterns

## Abstract

Generating high-quality crystals remains a bottleneck in biological and materials sciences. Here a counter-diffusion method was used to improve the X-ray diffraction quality of the N-terminal domain of *Drosophila melanogaster* Toll receptor crystals. It was observed that crystallization occurred with a peculiar pattern along the capillary resembling Liesegang bands; this phenomenon is described at both macroscopic and atomic levels. It was found that bands appeared for native protein as well as for co-crystals of magic triangle (I3C)-bound protein even though they crystallize in different space groups. Crystallization occurred with a linear recurrence independent of the precipitant concentration and a protein-specific spacing coefficient. Bandwidth varied along the capillary, oscillating between large precipitation areas and single crystals. The reported data suggest that repetitive patterns can be generated with biological macromolecules in the presence of sodium malonate as a crystallization agent. A comparison with typical Liesegang patterns and the possible mechanism underlying this phenomenon are discussed.

## Abstract 2

The observation of a repetitive pattern obtained for protein crystals formed by reaction–diffusion in capillary tubes is reported for the first time.

## 1. Introduction   

Repetitive patterns such as the stripes of a zebra or those of agate rocks – although very common in nature – are generated by complex mechanisms that are still only partially understood and remain difficult to model mathematically. There is no unifying theory that explains this widespread phenomenon that has intrigued scientists over more than a century. An early description of repetitive pattern formation by Raphael E. Liesegang focused on the chemical reaction occurring in gels between counter-diffusing electrolytes (Liesegang, 1896 ▶). Liesegang noticed that stratification of parallel precipitation bands originates from anions (outer electrolyte) diffusing in a capillary containing a gel impregnated with cations (inner electrolyte). The formation of bands resulting from the precipitation of the weakly soluble salt occurs rhythmically, and these bands are separated by clear gel instead of forming a continuous precipitation zone, as one would have expected.

Wilhelm Ostwald was the first one to propose an explanation to the phenomenon (Ostwald, 1897 ▶, 1925 ▶). His hypothesis is based on the propagation of a supersaturation wave and belongs to the pre-nucleation theories, in contrast to more recent post-nucleation theories, in which repetitive patterns are produced in essentially homogeneous and continuous colloid. All theories use equations derived from Fick’s law of diffusion. In a nutshell, molecular transport is driven by the initial difference of concentrations of the reagents in the gel. Diffusion is counteracted by the principle of neutrality and the change of solubility of the reaction product that precipitates upon reaching its solubility limit. This in turn depletes the reagents and sets off another wave of diffusion. The difference in the chemical potential during the reaction is the driving force for nucleation and crystal growth. The post-nucleation theories include a sink term in the diffusion equation to model this effect, in contrast to the pre-nucleation ones, where it is an assumption. The additional term refers to the evolution of the reagents’ concentrations over time, and in some cases also takes into account the physico-chemical properties of the ion product. Following the law of mass action, precipitation at a given position leads to a local decrease in supersaturation. This explains why precipitation zones stop growing. While the precipitate is trapped in the network of the gel the front of migration propagates the reagents. After a while at a given distance their concentration will be high enough to react again, thus explaining the alternation of clear areas and bands of precipitation along the gel. The two counter-diffusing reagents are gradually consumed and produce rings of increased spacing as a function of time.

Liesegang patterns are routinely described by four laws: (i) the spacing law (Morse & Pierce, 1903 ▶); (ii) the time law (Jablczinsky, 1923 ▶); (iii) the width law; and (iv) the Matalon–Packter law (Matalon & Packter, 1955 ▶). The first law predicts the spacing between consecutive bands, which follows a geometric series where *X*_*n*_/*X*_*n*+1_ = *P*, with *X* being the position of a given band (*n*) from the gel surface and *P* the so-called spacing coefficient. The time law, *X*_*n*_ ≃ *t*_*n*_^1/2^, gives the relation between the distance of a band and the square root of its time of appearance *t*_*n*_. The width law is similar to the spacing law in that it predicts that the width of consecutive bands also follows a geometric series with *w*_*n*_/*w*_*n*+1_ = *Q*, where *Q* is the width coefficient. The Matalon–Packter law shows that the spacing coefficient is not a universal quantity but depends instead on the initial concentrations of the electrolytes: *P* = *F*(*b*_0_) + *G*(*b*_0_)/*a*_0_, where *a*_0_ and *b*_0_ are the initial concentrations of the outer and inner electrolytes, respectively, and *F* and *G* are decreasing functions of their arguments.

Although an experimental setup similar to Liesegang’s experiments in gel has been used for almost two decades for protein crystallization by the method of counter-diffusion in capillaries (García-Ruiz *et al.*, 2001 ▶; Ng *et al.*, 2003 ▶; Otálora *et al.*, 2009 ▶), repetitive patterns have not been observed for proteins before. Here we report, for the first time, on such an occurrence for crystals of the N-terminal domain of the *Drosophila melanogaster* Toll receptor using sodium malonate as a crystallization agent. Toll is a transmembrane glycoprotein that functions as a cytokine receptor and is critically involved in both embryonic development and immunity in fruit flies. It is the founding member of an important family of germ-line-encoded pathogen pattern-recognition receptors in mammals, the Toll-like receptors (TLRs) (Gay & Gangloff, 2007 ▶). Previously we determined low-resolution structures of the receptor’s extracellular domain in the presence and absence of its ligand Spätzle by electron microscopy (Gangloff *et al.*, 2008 ▶). In contrast, X-ray crystallographic studies have been hampered by the heterogeneity of the system undergoing negative cooperativity (Weber *et al.*, 2005 ▶). We present the method used to generate material suitable for such a study and note the unusual behaviour of Toll crystals obtained by counter-diffusion crystallization in capillaries in the presence of malonate.

## 2. Experimental   

### 2.1. Protein production and crystallization   

**Figure 1:**
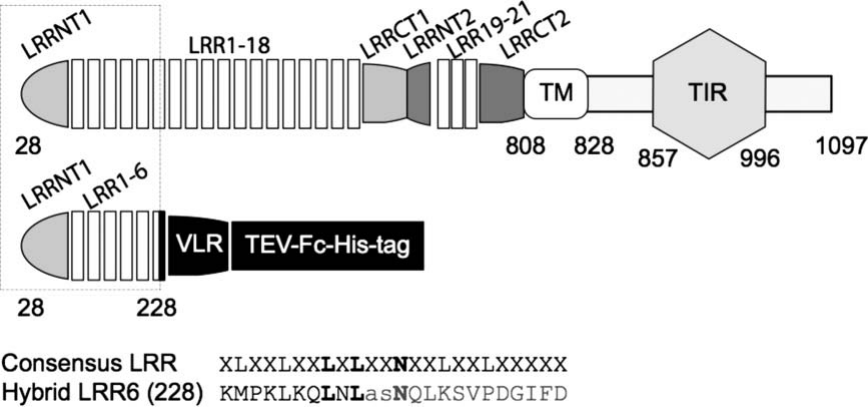
Baculovirus expression construct. *Drosophila melanogaster* Toll receptor is a 1097-residue-long transmembrane protein with an ectodomain of leucine-rich repeats (28–801 residues), a helical transmembrane region (TM, residues 808–828) and an intracellular domain with a Toll-interleukin-1 receptor signalling domain (TIR domain, residues 857–996). The N-terminal region of the Toll ectodomain Met1–Leu228 is expressed as a chimera with the VLR C-terminal capping structure Asn133–Thr201, a TEV protease cleavage site and the constant fragment of human immunoglobulin G1 for purification (Gangloff *et al.*, 2013 ▶). The 24 residue-long consensus LRR sequence is given with *X* standing for any amino acid, L for Leu and N for Asn. The sequences of Toll and VLR are fused in the middle of the conserved leucine-rich repeat motif of LRR6 at Leu228.

The N-terminal domain of the Toll receptor (Met1–Leu228, referred to as Toll_N6_ in reference to the presence of the six first leucine-rich repeats of Toll) was expressed as a chimera receptor with residues Asn133–Thr201 of the variable lym­phocyte receptor (VLR) following the leucine-rich repeat (LRR) hybrid technique (Jin & Lee, 2008 ▶). The construct also contained a cleavable fusion with the constant fragment of human immunoglobulin G1 (Fc) to facilitate purification (Fig. 1 ▶). Expression was carried out in a baculovirus/insect cell system (Bac-to-Bac, Invitrogen). The protein of interest was purified from the cell culture supernatant by protein A affinity chromatography (GE Healthcare). The Fc fusion was cleaved by tobacco etch virus (TEV) protease (Kapust *et al.*, 2001 ▶). The digestion products were separated by protein A affinity and Toll_N6_–VLR was then further purified by size exclusion chromatography (GE Healthcare) in a buffer containing 100 m*M* sodium chloride, 20 m*M* Tris–HCl pH 7.0. Monomers of Toll_N6_–VLR (approximately 35 kDa, pI = 8.5) were concentrated to 20 mg ml^−1^ (0.6 m*M*) and submitted to crystallization trials in sitting drops using the vapour-diffusion method. An initial hit was obtained in a condition of the JCSG-plus Screen (Qiagen), which contains 2.4 *M* sodium malonate pH 7.0.

### 2.2. Dynamic light scattering   

Dynamic light scattering (DLS) was used to determine the diffusion coefficient of Toll_N6_–VLR in its conditioning buffer 100 m*M* NaCl, 20 m*M* Tris–HCl pH 7.0, using a Zetasizer Nano-S instrument (Malvern). Protein samples at various concentrations between 0.2 and 20 mg ml^−1^ (6–600 µ*M*) were centrifuged for 5 min at 13 000*g* to remove any aggregates. Aliquots of 40 µl were loaded into disposable solvent resistant micro cuvettes (Malvern), and ten DLS measurements were made, taken in triplicate and averaged. The diffusion coefficient *D* that relates to the Brownian motion of the molecules in solution is proportional to the exponential decay of the scattering intensity. *D* is calculated by fitting the autocorrelation curve of a monodisperse sample to an exponential function.

### 2.3. Vapour diffusion crystallization   

Sitting drops and hanging drops were set up manually but failed to reproduce the initial hit. Systematic screening (Grid Screen Sodium Malonate, Hampton Research, and manual screens) revealed that Toll_N6_–VLR crystals could be obtained at pH 7.0 in the presence of 1.5–1.8 *M* sodium malonate with protein concentrations ranging from 2 to 20 mg ml^−1^ (0.06–0.6 m*M*).

### 2.4. Counter-diffusion crystallization   

For counter-diffusion crystallization in agarose gels, a mixture of 10 µl of Toll_N6_–VLR protein at 20 mg ml^−1^ with 10 µl of agarose 0.6% was boiled for 1 min and cooled down to about 313 K before use. The protein mixture (8 µl) was introduced into capillary tubes of internal diameter 0.3 mm and length 80 mm (Capillary Tube Supplies Ltd). It solidified within a few minutes, upon which sodium malonate pH 7.0 (20 µl) at a given concentration between 2.4 and 3.4 *M* was added on top of the gel. The capillary was sealed at both ends using plasticine and nail polish.

The counter-diffusion method was also used for heavy metal derivatization. The magic triangle, 5-amino-2,4,6-triiodoisophthalic acid (I3C), was successfully incorporated into the protein by adding the following solution onto the gel: 0.15 *M* I3C, 2.89 *M* sodium malonate pH 7.0.

Egg-white lysozyme (14.3 kDa, pI = 11.35; Sigma) was dissolved in 100 m*M* NaCl, 20 m*M* Tris–HCl pH 7.0 at concentrations between 20 and 100 mg ml^−1^ (1.4–7 m*M*). Capillaries of gelled lysozyme solutions at 0.3% agarose were set up against a reservoir of 3.4 *M* sodium malonate pH 7.0. Measurements of crystallization zones along the capillaries were taken after two weeks of diffusion at 291 K as for Toll_N6_–VLR.

### 2.5. Crystal extraction and cryocooling   

Sodium malonate is cryoprotectant at 2.4 *M* and higher concentrations at pH 7.0. In the counter-diffusion method we estimated that the precipitant is evenly distributed at 71.4% of its initial concentration after a couple of weeks. The initial concentration of 3.4 *M* sodium malonate thus generates a final concentration of 2.4 *M* once equilibrium is reached. Lower initial concentrations were used as well (2.4 and 2.89 *M* sodium malonate supplemented with increasing concentrations of I3C). In order to reach concentrations that were cryoprotectant, a second round of diffusion was allowed to take place by opening the top of the capillaries and replacing the reservoir solutions with 10 µl of precipitant stock solution (3.4 *M* for native protein and 2.89 *M* supplemented with I3C in the derivatization experiment). After another couple of weeks, the malonate concentrations were cryoprotectant for all conditions tested and reached values between 2.6 and 2.9 *M* for initial concentration ranging from 2.4 to 3.4 *M* solutions, respectively.

Crystals were extracted from the capillaries, dissected away from the gel and flash-frozen using malonate at the predicted concentrations as a cryoprotectant agent. The I3C-derivatized crystals were submitted to an intermediate washing step to remove nonspecifically bound I3C, which could decrease the anomalous differences of the crystal. To achieve this, crystals were back-soaked in mother liquor containing 2.8 *M* sodium malonate pH 7.0 in hanging drops left to equilibrate for an hour against a reservoir of 500 µl of the same solution.

### 2.6. Data collection, phasing and model refinement   

The anomalous signal of the bound iodine was exploited for phase determination using the single anomalous dispersion method. Oscillation images were integrated and reflection intensities were merged and scaled using the *XDS* package (Kabsch, 2010 ▶). In the *Phenix Autosol* package *Xtriage* analysis revealed that a data set encompassing the first 180° oscillation provided an anomalous signal at 4.6 Å (up to 4.0 Å, from a more optimistic point of view) (Adams *et al.*, 2010 ▶). The positions of the iodine sites were identified by HYSS analysis with a figure of merit of 0.32 and a Bayes correlation coefficient of 32 ± 4. Electron density examination in *Coot* (Emsley & Cowtan, 2004 ▶) confirmed the hand of the tetragonal space group and allowed manual building of 85% of the molecular model. The partial model was then used for molecular replace­ment in the 2.4 Å-resolution native orthorhombic data set. The final models were obtained after numerous rounds of refinement in *Buster* (Blanc *et al.*, 2004 ▶) and manual re-building in *Coot*. Both 2|*F*_o_|−|*F*_c_| and |*F*_o_|−|*F*_c_| electron density maps were used in model building. TLS refinement was used with one group per chain. A total of 323 water molecules that were within hydrogen-bonding distance of the protein were added to the native structure. Both structures were assessed for correctness and validated using *Molprobity* (Chen *et al.*, 2010 ▶); these structures have been deposited in the Protein Data Bank (Berman *et al.*, 2000 ▶) with the accession codes 4arn and 4arr, for the native and the I3C-bound proteins, respectively. Crystallographic data are summarized in Table 1 ▶.

## 3. Results and discussion   

### 3.1. Counter-diffusion to improve crystal diffraction quality   

In this study we used the N-terminal domain of Toll encompassing the 201 first amino acids produced as a VLR hybrid protein, a strategy that can generate stable deletion constructs established for mammalian TLRs (Jin *et al.*, 2007 ▶; Kang *et al.*, 2009 ▶; Kim *et al.*, 2007 ▶; Yoon *et al.*, 2012 ▶). The initial crystals obtained using a vapour diffusion method at 2.4 *M* malonate pH 7.0 were not reproducible and diffracted to a maximum resolution of 7–15 Å. Lower malonate concentrations gave reproducible conditions that required cryoprotection to avoid ice formation upon cooling. The additional handling often resulted in crystal damage. Indeed crystals soaked in 2.4 *M* sodium malonate (which is the lowest concentration that provides cryoprotection at pH 7.0) or in lower amounts (*i.e.* the concentration of the mother liquor supplemented with other cryoprotectants such as glycerol and ethylene glycol) tended to split.

**Figure 2:**
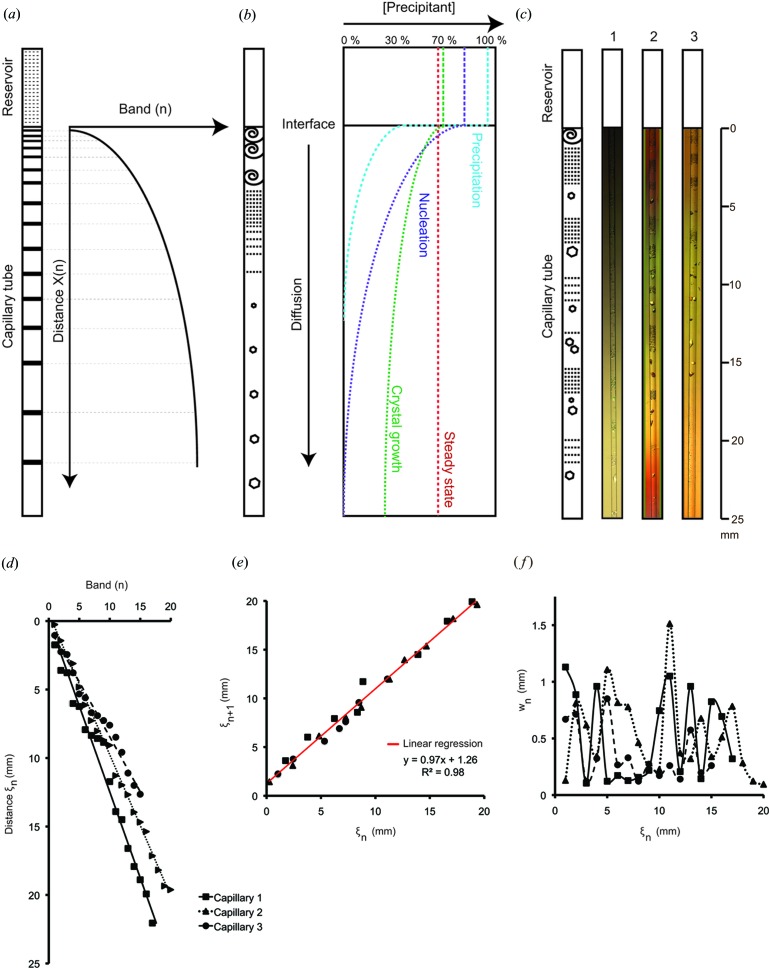
Different precipitation scenarios produced by counter-diffusion in capillaries. (*a*) Liesegang bands formed by minerals. The experimental setup is represented by a reservoir of highly concentrated anions and a capillary tube filled with a homogenous gel containing cations. Left panel: graphical representation of the band formation along the capillary. The two counter-diffusing reagents are gradually consumed upon precipitation of their ion product, which generates rings of increased spacing as a function of time. (*b*) Protein crystallization by counter-diffusion in capillaries. The capillary contains a small volume of gelled protein solution at millimolar concentration and the reservoir contains a large volume of precipitant at molar concentration. Initially the precipitant penetrates the gel and accumulates at its highest concentration near the interface, where it triggers protein precipitation. This achieves the highest supersaturation level and ratio, which is illustrated with a blue line on the graph. As the precipitant equilibrates against the capillary, lower concentrations are reached gradually inside the gel (purple, green and red lines, respectively) and both the levels and the ratios of supersaturation decrease in time and space, which is favourable for nucleation and crystal growth. The steady state is reached in approximately two weeks. (*c*) Liesegang-like pattern for Toll_N6_–VLR in the presence of malonate. Capillary 1 was left to equilibrate against 2.4 *M* sodium malonate pH 7.0; capillary 2 against 3.4 *M* sodium malonate pH 7.0; and capillary 3 against 0.15 *M* I3C, 2.89 *M* sodium malonate pH 7.0. Photographs of the lower section of each capillary have been taken after two weeks. (*d*) Graph representing the relative distances ξ_*n*_ of Toll_N6_–VLR bands as a function of the number of bands in each capillary. (*e*) Determination of the spacing coefficient *P*, represented by the slope of the linear regression (*P* = 0.97 for Toll_N6_–VLR). The graph shows the relative distances of successive bands ξ_*n*+1_ represented as a function of ξ_*n*_, from all Toll_N6_–VLR capillaries. (*f*) Toll_N6_–VLR does not obey the width law. The graph shows the relative distance of band *n* (ξ_*n*_) represented as a function of its width (*w*_*n*_).

We then attempted the counter-diffusion method using the capillary technique (García-Ruiz & Moreno, 1994 ▶; Otálora *et al.*, 1999 ▶; Ng *et al.*, 2003 ▶; García-Ruiz *et al.*, 2001 ▶; Sauter *et al.*, 2001 ▶). This technique was inspired by the Liesegang experiments with minerals that diffuse in a gel in the absence of convection (Fig. 2 ▶ *a*). Briefly the precipitant is added in excess at different concentrations on top of a capillary filled with gelled protein solution, sealed at both ends and left to equilibrate. In such a setup the precipitant diffuses along the capillary and reaches a steady state equilibrium within a couple of weeks according to its diffusion rate (size). The protein in contrast does not diffuse significantly on the same time scale and encounters instead the supersaturation wave generated by the precipitant concentration gradient. The difference in chemical potential between supersaturated and soluble proteins leads to a change of phase of the protein with precipitation or crystallization, depending on the level and the rate of supersaturation (García-Ruiz, 2003 ▶).

The highest precipitant concentration is found initially at the top of the capillary (Fig. 2 ▶ *b*). This leads to an area of heavy precipitation in the vicinity of the interface where the rate of supersaturation is at its highest. Further down the capillary, supersaturation decreases as a function of precipitant concentration and leads to nucleation in the labile zone and crystal growth in the metastable zone, according to the phase diagram. Using malonate concentrations at 2.4 *M* and higher, we found that this method yielded crystals with suitable diffraction power (resolution ranging from 2.4 to 3.0 Å). It is likely that this method is more successful because of the slower crystallization process and the transport properties (diffusion controlled transport), which mean that crystallization occurs over weeks instead of days for vapour diffusion.

The derivative crystals diffracted to a much lower resolution (3.0 Å for the best data set) than the native ones (best at 2.4 Å). It is possible that the agarose ‘coating’ of the crystals formed a protective layer that prevented crystal damage during handling of the native crystals. According to their respective solvent content of 62 and 59% for the native and derivative crystals, the latter were expected to diffract better. Either incomplete substitution or the additional handling during back-soaking was detrimental to the quality of the derivative crystals.

### 3.2. Native and derivative crystals form a repetitive pattern along the capillary   

When the precipitant solution was added onto the gelled protein compartment in the capillary tubes, its diffusion in the absence of convection triggered the periodic apparition of areas of precipitation and crystallization (Fig. 2 ▶ *c*). This was unexpected, as the phenomenon of pattern formation has only been described for minerals so far. Proteins have a narrow window of conditions able to overcome the energy barrier of nucleation and this is thought to prevent the formation of bands (García-Ruiz *et al.*, 2001 ▶).

In contrast our Toll construct crystallized in bands over a couple of weeks. Initially heavy precipitation was apparent, starting from the interface between the reservoir and the gel, and precipitation progressed at a rate of 1.8 × 10^−6^ cm^2^ s^−1^ along the capillary until it faded (supplementary material movie S1^1^). After a few days bands of nucleation appeared further down the capillary, and after a couple of weeks they became more discernable as crystal growth occurred. It is intriguing that the wave of precipitation is much slower than the diffusion coefficient of sodium malonate at 8.45 × 10^−6^ cm^2^ s^−1^. According to dynamic light scattering experiments the diffusion coefficient of our protein of interest is ten-times lower at 1.8 × 10^−7^ cm^2^ s^−1^ (samples in conditioning buffer had a *Z*-average size of 12.7 nm and a polydispersity index of 0.1).

Next we characterized the pattern formation of Toll crystals by measuring the distance between bands and their bandwidth under the microscope (Fig. 2 ▶ *d*). We have not recorded the distances from the interface to the band (distance *X*) but decided instead to measure the band positions according to George and Varghese’s moving boundary theory for improved accuracy (George & Varghese, 2002 ▶). Thus, measurements were taken from the lower limit of the first band to give the relative distance ξ of the following ones.

The band distribution along the capillary was found to be periodic and best described mathematically with a linear regression. A variable number of bands appeared as a function of the concentration of precipitant and the presence of the magic triangle, I3C, used for phasing the structure (Fig. 2 ▶ *d*). Surprisingly the spacing coefficient, determined using the relative distances of successive bands ξ_*n*+1_ represented as a function of ξ_*n*_, is conserved regardless of the initial conditions of the reservoir and is found to be close to 1.0 (Fig. 2 ▶ *e*). This result seems to suggest that our system obeys the spacing law but fails to follow the Matalon–Packter law assuming that the range of concentrations tested was meaningful (Antal *et al.*, 1998 ▶).

Oscillations in bandwidth (*w*_*n*_) were noticed in conjunction with the relative distance (ξ_*n*_) of a given band (Fig. 2 ▶ *f*). Both the amplitude and the frequency of the bands vary in undetermined ways. We found that, in contrast to the spacing law of repetitive pattern formation, the width law does not apply within the time scale of the experiment. The fluctuations in bandwidth are probably linked to the mechanism of Oswald ripening, with large crystals growing at the expense of smaller ones that dissolve. In other words we suspect that, although the precipitant has diffused along the capillary in the time scale of the experiment, the nucleation and crystal growth are slower procedures that were still in progress when the experiments were stopped. However, large crystals were obtained in different areas of the capillary and amid precipitation areas as if the protein reacted immediately to the wave of supersaturation along the capillary.

In order to form a repetitive pattern our protein of interest probably displays faster reaction kinetics than other proteins previously studied using this method. This hypothesis is supported by the recorded speed of propagation of the precipitation front. Alternatively the precipitant agent malonate might be responsible or the combination of both malonate and Toll protein. Moreover there was no obvious correlation between the position of a given crystal in the capillary and its diffraction power, suggesting that each periodic area provides a window of supersaturation levels for screening the entire crystallization parameter space.

### 3.3. Periodic pattern formation is independent of crystal packing and the precipitant binding mode of the protein   

**Figure 3:**
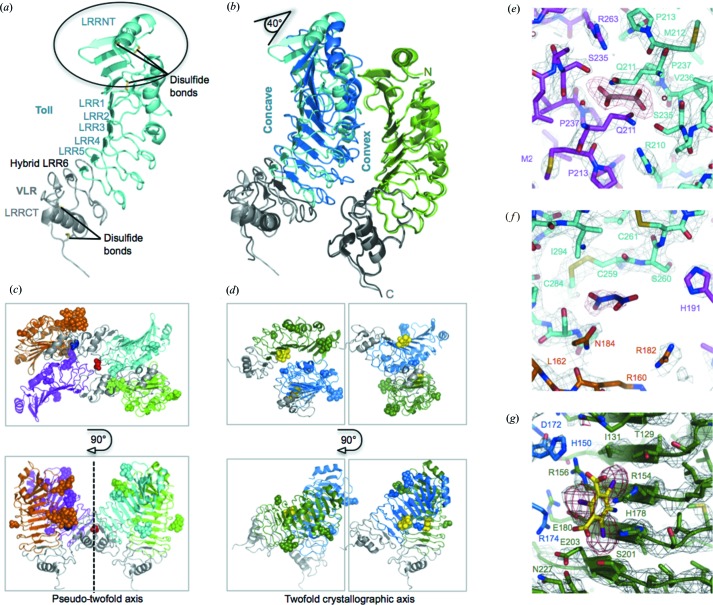
Atomic details of the native and derivative Toll_N6_–VLR crystal structures. (*a*) Crystal of the N-terminal domain of the Toll receptor. The six LRRs are capped with disulfide-rich regions at the N- (LRRNT) and C-terminus (LRRCT). Toll is represented in cyan and VLR in grey. The fusion occurs at LRR6. (*b*) Building blocks of the crystal structures. Native and derivative crystals both contain head-to-head pairs of Toll_N6_–VLR molecules interacting at their concave–convex interface. Upon superposition of the green molecules (light green from the native structure, dark green from the derivative one) it appears that the arrangement of the blue molecules is shifted by 40° to accommodate binding of the magic triangle I3C between the concave and the convex sides in the derivative structure. (*c*) Packing organization of the native structure. The native structure contains four molecules per asymmetric unit, with two pairs linked by a pseudo-twofold axis of symmetry. Top and side views are depicted. Glycan structures are represented as spheres coloured according to the Toll chain that they relate to. The VLR fusion is shown in grey. Malonate ions (in red or blue) are located at the right flank of the leucine-rich motif of the glycoprotein in its native state. (*d*) Higher-order symmetry of the derivative structure. Toll bound to I3C has a tetragonal symmetry, compared to the orthorhombic one of the native form. It only has two molecules per asymmetric unit. Two adjacent asymmetric units are shown to highlight the difference in crystal packing. The different views are given in the same orientation in (*c*)–(*d*) and asymmetric units have been boxed to show the differences between the crystal forms. (*e*)–(*f*) Binding modes of malonate in the native structure. (*g*) Binding of I3C in the derivative structure. I3C is represented in yellow at the concave side of Toll. The 2*F*_o_−*F*_c_ map is shown in grey contoured at a level 3 and the difference map is shown in red at a level 5. Figure adapted from Gangloff *et al.* (2013 ▶).

Upon structure determination using a combination of single-wavelength anomalous dispersion of the iodinated species and molecular replacement the atomic structure of the protein could be analysed and the crystal contacts revealed (Fig. 3 ▶; Gangloff *et al.*, 2013 ▶). The atomic structures were determined for both native and derivative forms at 2.4 and 3.0 Å, respectively. Toll_N6_–VLR adopts an arc shape typical of the leucine-rich repeat proteins with an elongated N-terminal cap (LRRNT) that adopts a new fold (Fig. 3 ▶ *a*; Gangloff *et al.*, 2013 ▶). Surprisingly native and derivative crystals were found to belong to different space groups even though they had similar bipyramidal morphologies. The former belong to the orthorhombic space group *P*2_1_2_1_2_1_ with four molecules in the asymmetric unit (Fig. 3 ▶ *c*), whereas the latter crystallized in the tetragonal space group *P*4_3_2_1_2 with two molecules in the asymmetric unit (Fig. 3 ▶ *b*).

Although Toll_N6_–VLR is monomeric in solution (Gangloff *et al.*, 2013 ▶), both native and derivative lattices involve similar arrangements of head-to-head protein pairs with concave–convex interactions (Fig. 3 ▶ *b*). However, I3C binding at the concave side of each protein, interacting with charged residues of the leucine-rich repeat region, modifies the packing of the second molecule by tilting it along its long axis by approximately 40° compared to the native structure. This in turn increases the crystal symmetry. The two pairs of Toll_N6_–VLR molecules that are related to each other by a pseudo-twofold axis (Fig. 3 ▶ *c*) in the native structure are now linked by a crystallographic axis of symmetry (Fig. 3 ▶ *d*). Overall the packing of I3C-bound Tolls involves mainly protein–protein contacts whilst the native structure displays more glycan contacts both within and outside of the asymmetric unit (Figs. 3 ▶ *c*–3 ▶ *d*).

The fact that the protein crystallized in two different space groups suggests that the Liesegang-like phenomenon is independent of crystal packing. Each molecule involved in the crystallization process is also responsible for the formation of the repetitive pattern in a colligative manner. Here we show that bands occur regardless of the binding mode of the ionic species at play. Indeed crystallization buffer molecules could be ascribed in the electron density of both the native and derivative structures and they make contacts at different areas of the glycoprotein (Figs. 3 ▶ *e*–3 ▶ *g*; Gangloff *et al.*, 2013 ▶). A first malonate ion is located at the pseudo-twofold axis of symmetry in the native structure (Fig. 3 ▶ *e*). A second one is observed in the vicinity of the concave–convex interface (Fig. 3 ▶ *f*). Both molecules interact with the right flank of the LRR motif. In contrast molecules of I3C make extensive ionic interactions at the concave side of Toll as mentioned earlier (Fig. 3 ▶ *g*).

In contrast to the depletion phenomenon obtained with typical Liesegang experiments, the crystallization agents are in large excess, and although we observe direct contacts between them and the protein, their concentration would not be significantly reduced in this experimental setup.

### 3.4. Repetitive patterns are obtained with lysozyme   

**Figure 4:**
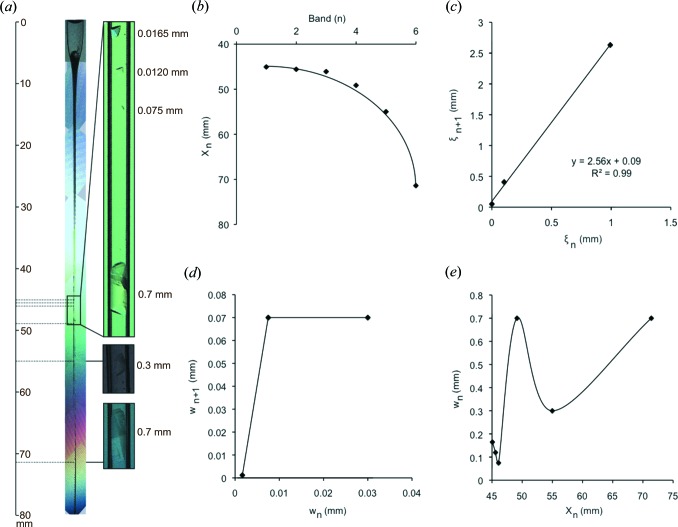
Periodicity in lysozyme crystallization in the presence of malonate. (*a*) A gelled lysozyme capillary (50 mg ml^−1^, 3.5 m*M*) equilibrated against a reservoir of 3.4 *M* sodium malonate pH 7.0. Pictures are taken after a couple of weeks of diffusion. (*b*) The crystallization periodicity of lysozyme is not linear but follows a geometric series as predicted by Liesegang. (*c*) Determination of the spacing coefficient of lysozyme (*P* = 2.56). (*c*) Lysozyme does not obey the width law. (*d*) As for Toll_N6_–VLR, the bandwidth of lysozyme follows a periodic function.

As repetitive patterns had never been described before for proteins, we wondered whether the effect was protein specific or due to sodium malonate. We are not aware of any previous report of sodium malonate being used in counter-diffusion experiments. In order to check this hypothesis we decided to set up capillaries with egg-white lysozyme (Sigma) in exactly the same conditions as Toll_N6_–VLR. Briefly, lyophilized lysozyme was dissolved in 100 m*M* NaCl, 20 m*M* Tris–HCl pH 7.0 at increasing concentrations up to 100 mg ml^−1^ (7 m*M*). Capillaries of gelled protein solution at 0.3% agarose were set up against a reservoir of 3.4 *M* sodium malonate pH 7.0. As for Toll_N6_–VLR, lysozyme is positively charged at neutral pH. In addition it had been reported to crystallize in the presence of malonate in vapour diffusion experiments (McPherson, 2001 ▶). We managed to obtain crystals of lysozyme by counter-diffusion that reached sizes up to 0.7 mm in a couple of weeks (Fig. 4 ▶ *a*). The crystals diffracted to a resolution of 1.8 Å in-house. They were tetragonal (*P*4_3_2_1_2) with unit-cell parameters of *a* = *b* = 78.59, *c* = 38.13 Å, one molecule per asymmetric unit and a solvent content of 40%.

There was no precipitation area among the single crystals of lysozyme. We went on to characterize the location (both relative distance ξ_*n*_ and the absolute distance *X*_*n*_ were measured, the latter is shown in Fig. 4 ▶) and found that, in contrast to Toll_N6_–VLR, lysozyme crystallization followed a geometric series with a scaling coefficient of about 2.5 (Figs. 4 ▶ *b*–4 ▶ *c*). It is noteworthy that only a 1000-fold molar excess was used for lysozyme instead of a 10 000-fold excess of malonate in the case of Toll_N6_–VLR, which might explain why lysozyme bands plateaued. However they also failed to obey the width law and showed an oscillatory behaviour as a function of the distance from the interface between the reservoir and the protein gel (Fig. 4 ▶ *d*). Here again large crystals could be obtained at different areas along the capillary, which suggests a nonlinear behaviour in crystal growth kinetics.

## 4. Conclusion   

In this work we have shown that biological macromolecules are not exempt from forming repetitive patterns by reaction–diffusion. To a first approximation, it appears that the truncated Toll construct does not obey the fundamental laws of periodic precipitation. However, given the results obtained using lysozyme and malonate, it is plausible that our experiments with Toll are biased by the differences in concentrations between precipitant (outer electrolyte) and protein (inner electrolyte), the length of the capillary, and, additionally, by the time scale of the experiment (to account for Ostwald ripening). Our results strongly suggest that the spacing coefficients are protein specific and, more importantly, that the mechanisms underlying protein crystallization are still not fully understood as this phenomenon was not predicted by available theories. The role that malonate plays in the process is particularly puzzling as this kosmotropic anion can bind the protein, neutralize its positive charge and trigger supersaturation by a salting-out effect. A recent development in monitoring capillaries by *in situ* dynamic light scattering (Oberthuer *et al.*, 2012 ▶) will be invaluable in further characterizing the nontrivial process of reaction–diffusion for protein crystallization.

## Supplementary Material

Movie S1: progression of the precipitation front. Four TollN6-VLR capillaries were set up with reservoirs either of 3.4 M sodium malonate pH 7.0 (two capillaries on the left) or 0.15M I3C, 2.89M sodium malonate pH 7.0 (two capillaries on the right). A picture was taken every hour over ten days. A movie was generated with 24 frames per second. The progression of the precipitation front is clearly visible in the second capillary from the left.. DOI: 10.1107/S0021889812051606/he5568sup1.mov

## Figures and Tables

**Table 1:** Crystallographic data

	Native	I3C Derivative
Data collection		
Space group	*P*2_1_2_1_2_1_	*P*4_3_2_1_2
Cell dimensions		
*a*, *b*, *c* ()	88.79, 93.28, 225.34	87.64, 87.64, 220.74
, , ()	90.0, 90.0, 90.0	90.0, 90.0, 90.0
Resolution ()	29.92.41 (2.542.41)†	47.403.00 (3.163.00)†
*R*_merge_ ‡	0.056 (0.542)†	0.138 (0.653)†
*I*/(*I*)	20.6 (3.0)†	14.1 (3.4)†
Completeness (%)	99.3 (97.1)†	99.9 (100.0)†
Redundancy	6.6 (6.0)†	13.0 (12.0)†

Refinement		
Resolution ()	29.92.4	46.73.0
No. of reflections	72763	17997
*R*_work_ (%)§	20.09	22.42
*R*_free_ (%)¶	21.58	25.39
No. of atoms	9283	4414
Protein	8715	4340
Heterogen atoms	245	74
Water molecules	323	0
*B* factor (^2^)	66.68	64.40
R.m.s. deviations		
Bond lengths ()	0.008	0.007
Bond angles ()	0.97	0.93
Cruickshank DPI		
based on *R*_work_ ()	0.246	2.408
Cruickshank DPI		
based on *R*_free_ ()	0.185	0.375

† Numbers in parentheses refer to the highest resolution shell.

‡ *R*_merge_= _*hkl*__*i*_|*I_i_*(*hkl*)*I*(*hkl*)|/_*hkl*__*i*_ *I*_*i*_(*hkl*), with *I_*i*_*(*hkl*) the intensity of an individual measurement of the reflection with Miller indices *h*, *k* and *l*, and *I*(*hkl*) the mean intensity of that reflection. Value calculated for *I*3(*I*).

§ *R*_work_= _*hkl*_[||*F*_obs_(*hkl*)| 1|*F*_calc_(*hkl*)||]/|*F*_obs_(*hkl*)|, where |*F*_obs_(*hkl*)| and |*F*_calc_(*hkl*)| are the observed and calculated structure factor amplitudes.

¶ *R*_free_ is calculated as *R*_work_ with 5% reflections omitted from the refinement process.

## Footnotes

1 The movie S1 discussed in this paper is available from the IUCr electronic archives (Reference: HE5568). Services for accessing this material are described at the back of the journal.
